# Reliability of Fractional Flow Reserve in Non-Infarct-Related Arteries in ST-Segment Elevation Myocardial Infarction Patients Undergoing a Pharmaco-Invasive Approach

**DOI:** 10.7759/cureus.52668

**Published:** 2024-01-21

**Authors:** Ayush Shukla, Sudhanshu K Dwivedi, Sharad Chandra, Gaurav Chaudhary, Akhil Sharma, Rishi Sethi, Akshyaya Pradhan, Pravesh Vishwakarma, Monika Bhandari, Abhishek Singh

**Affiliations:** 1 Cardiology, King George's Medical University, Lucknow, IND

**Keywords:** angioplasty and stenting, st-segment elevation myocardial infarction (stemi), fractional flow reserve (ffr), coronary stenosis, coronary revascularization

## Abstract

Objectives

We investigated the reproducibility of fractional flow reserve (FFR) of significant stenoses (≥70% narrowing) in the non-infarct related artery (NIRA) during the pharmaco-invasive percutaneous coronary intervention (PCI) in patients with ST-segment elevation myocardial infarction (STEMI) within 24 hours of thrombolysis and at a follow up of 2-3 weeks.

Background

STEMI with multivessel disease has worse outcomes. The benefits of FFR-directed PCI of NIRA at the time of primary PCI are yet controversial. Assessing the hemodynamic severity of the NIRA may help in deciding the management strategy of these lesions, save time, and avoid complications.

Methods

Thirty-one patients undergoing PCI for STEMI under a pharmaco-invasive approach were prospectively recruited. The FFR measurements in 34 stenoses (≥70% diameter stenosis) were obtained immediately after PCI of the culprit stenosis and were repeated at a mean follow-up of 17.6 ± 3.55 (14-21) days. In addition, time to thrombolysis, time from symptom onset to PCI, left ventricular ejection fraction (LVEF), quantitative coronary angiographic measurements of the non-culprit stenoses, and thrombolysis in myocardial infarction (TIMI) flow were recorded.

Results

There was a significant change in FFR values at follow-up as compared to baseline (0.78 ± 0.08 (0.68-0.93) to 0.77 ± 0.08 (0.67-0.93)) (p = 0.014). In four of the lesions, the FFR values differed by >0.05 at follow-up. The follow-up FFR values led to a change in the management strategy in 5 out of 31 patients (15%) of the lesions. The TIMI flow, percentage diameter stenosis, minimum lumen diameter, and LVEF did not change. There were no predictors of this change in FFR values.

Conclusions

During the acute phase of STEMI, the severity of non-culprit coronary artery stenoses can not be reliably assessed by FFR. The prolonged jeopardized state of myocardium in pharmaco-invasive PCI as compared to primary PCI seems to be responsible.

## Introduction

In developing countries like India, primary percutaneous coronary intervention (PCI) is possible only in a fraction of patients [1]. A large number of patients receive thrombolytic therapy and are then taken up for PCI based on a pharmaco-invasive strategy. Multivessel coronary artery disease is present in approximately one-half of patients with an acute MI [2-4], which is associated with a worse outcome [2]. Patients with significant stenosis in non-infarct related artery (NIRA) (>70% narrowing) should also be taken for PCI [3]. For the physiological significance of stenosis in intermediate lesions, the use of fractional flow reserve (FFR) has been widely implicated.

It is recognized that FFR values in the culprit vessel are higher during acute episodes when compared to measurements made after the microcirculation has had some time to recover [5,6]. Controversy persists on the use of FFR in acute coronary syndrome (ACS) settings with varying results [7,8].

The literature contains scarce data on the use of FFR in NIRA in ACS, ST-segment elevation myocardial infarction (STEMI), and no data on patients undergoing pharmaco-invasive PCI. Therefore, we felt it would be useful to comparatively analyze the values of FFR in patients who have ≥70% narrowing in NIRA and where the decision to intervene after 1-4 weeks has already been taken at the time of PCI to IRA. FFR was done at the time of index admission and reassessed at follow-up of about 1-4 weeks when patients were planned for non-culprit vessel PCI.

## Materials and methods

Study design

This was a single-center prospective observational study. Institutional Ethics committee, King George's Medical University ECR/262/Inst/UP/2013/RR-19 issued approval. Patients were enrolled between January 2016 and December 2016. Patients were included if all of the following criteria were met: age > 18 years, patients with STEMI who had received thrombolytic therapy and planned for PCI within 4-24 hours of index event in a stable hemodynamic condition with at least one more major epicardial artery/main vessel with diameter > 2.5 mm with angiographically significant stenosis (≥70%), which was planned for staged revascularization and willing to give informed consent for the index procedure and subsequent procedure if required.

Patients with age > 85 years, history of renal dysfunction, liver failure, cardiomyopathy, diffuse lesion in NIRA, left main disease, and history of prior CABG were excluded.

Protocol

Index Procedure

A coronary angiogram was performed within 4-24 hours of thrombolysis in patients with STEMI. The infarct-related artery (IRA) was identified on the basis of electrocardiographic, echocardiographic, and angiographic findings. PCI of the IRA was performed as per standard protocol. Thrombus aspiration, antiplatelet choice, stent implantation and type, predilation, and postdilation were left to the operator’s discretion. Lesion length, vessel diameter, and percentage diameter stenosis (% DS) as well as thrombolysis in myocardial infarction (TIMI) flow were noted in NIRA. Then if the patient continued to be in stable condition, NIRA was addressed. Preoperative left ventricular ejection fraction (LVEF) was noted on echocardiography.

FFR Measurement

The FFR wire was advanced distal to the stenosis in NIRA (Wire: Pressurewire Certus, 175 cm in length, St. Jude Medical Systems AB, Uppsala, Sweden).

Equalization was ensured and nitroglycerine intra-coronary bolus 100 mcg was given. FFR measurement was done in a steady state after a few seconds of bolus labeled as FFR I. Then intra-coronary adenosine 120 mcg was given and FFR measurement was done in a steady state labeled as FFR II which was the final FFR. The position of the sensor was recorded to ensure the same position of the FFR wire at follow-up. FFR was again measured and drift was recorded as per standard protocol. The FFR was automatically calculated by the instrument by dividing the mean distal coronary artery pressure by mean aortic pressure (Equipment: Radi Analyzer Express, St. Jude Medical Systems AB, Uppsala, Sweden) [9].

Follow Up

Between 1-4 weeks all patients were called up for staged revascularization of NIRA. LVEF by echocardiography and biochemical parameters were recorded and coronary angiogram was performed. Lesion characteristics of NIRA were noted. FFR was repeated by a standard protocol at the same position as noted in the index procedure. The treatment of NIRA was guided by this FFR. A value of ≤0.80 was considered a cut-off.

Endpoints 

The difference between mean FFR at the initial and follow-up procedures was noted.

Statistical analysis

Data was analyzed using IBM SPSS Statistics for Windows, Version 21 (Released 2012; IBM Corp., Armonk, New York, United States). Data has been represented as mean+SD for continuous variables and numbers (%) for categorical variables. The chi-square test, Fisher exact test, and independent samples "t" test have been used to compare the data. The correlation between baseline and follow-up parameters has been evaluated using the Pearson correlation coefficient and Blandt-Altman analysis. An "r" value > 0.7 depicts a strong correlation and a "p" value less than 0.05 indicates a statistically significant association. Clinical data between those with significant differences and those without was also compared.

## Results

Baseline characteristics

The study group comprised 31 patients with 34 lesions where two sets of FFR values were available for comparison. The mean vessel diameter in NIRA was 2.93 ± 0.24 mm (2.5-3.5 mm) with mean % DS being 78.2 ± 8.69.

All the patients were thrombolyzed with streptokinase. The mean duration of symptoms to thrombolysis was 4.96 ± 2.18 hours (2-11 hours) and the mean time to PCI from symptom onset was 18.29 ± 2.64 hours (10-23 hours).

After PCI of the IRA, TIMI flow grade 3 was achieved in 30 (96.7%) of the patients. There were three (9.6%) patients who received two stents in the IRA. The remaining 28 (90.3%) had a single stent in the IRA. All the stents were drug-eluting stents out of which 1 (3.2%) patient had an everolimus-eluting stent, 2 patients (6.4%) had a biolimus-eluting stent, and 31 patients (91.1%) had sirolimus-eluting stents. The mean ejection fraction was 48.7 ± 3.87% (38-56). GpIIb/IIIa antagonists were used in five patients (16.12%).

The mean lesion length in the NIRA was found to be 19.94 ± 5.4 mm (14-30 mm). The mean lesion diameter was 2.93 ± 0.24 mm (2.5-3.5 mm). The mean FFR values after adenosine in the NIRA at the time of PCI of IRA was 0.78 ± 0.08 (0.68-0.93).

The clinical, demographic, and angiographic characteristics of patients at baseline are shown in Table 1.

**Table 1:** Demographic, clinical, and angiographic profile of patients NIRA: Non-infarct-related artery; MI: Myocardial infarction

Characteristics	Number	Percentage
Mean age (years)	56.25 ± 7.71	
Male/female	22/9	71/29
Smoker	13	42
Tobacco chewer	16	52
Hypertension	17	55
Diabetes	10	32
H/O Cerebrovascular accident	5	16
Diagnosis		
Anterior wall (AWMI)	14	45.2
Inferior wall (IW) MI + Right ventricular MI	8	25.8
IWMI + Posterior wall (PWMI)	5	16.1
IWMI + PWMI + RVMI	2	6.4
IWMI	2	6.4
IRA		
Right coronary artery (RCA)	15	48.4
Left anterior descending coronary artery (LAD)	14	45.2
Left circumflex coronary artery (LCX)	2	6.4
NIRA		
LAD	13	38.2
LCX	12	35.2
RCA	5	14.7
Obtuse marginal (OM)	3	8.8
Diagonal (D1)	1	2.9

Follow up

Follow-up catheterization was performed after a mean duration of 17.6 ± 3.55 (14-21 days). The mean LVEF was 49.12 ± 4.07% (40-57%). LVEF did not change significantly in follow-up (p = 0.068). The mean NIRA lesion length and diameter remained at 19.94 ± 5.4 mm (14-30 mm) and 2.93 ± 0.24 mm (2.5-3.5 mm) respectively. The FFR value in the follow-up was 0.77 ± 0.08 (0.67-0.93). One of our patients had a difference of 0.02 from the initial value (0.81 to 0.79) but it resulted in a change in strategy from leaving the lesion on drugs to stenting it as the value was ≤0.8, which we had decided was the cut-off for stenting at the start of the study.

**Figure 1:**
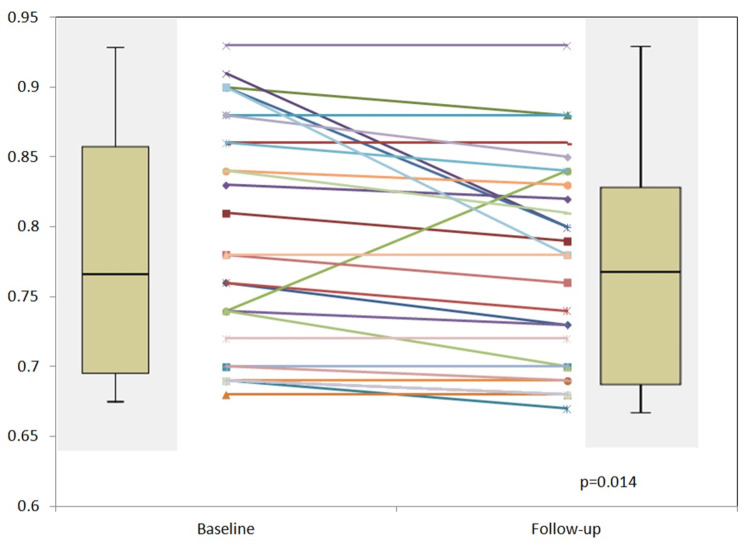
Plot of FFR values at baseline and at follow-up FFR: Fractional flow reserve

The change in the FFR values was found to be significant after statistical analysis (0.78 ± 0.08 (0.68-0.93) to 0.77 ± 0.08 (0.67-0.93)) (p = 0.014). The numerical difference in the mean value was however 0.01 (Figure 1).

We found a significant difference in 4 (11.7%) of our initial values as compared to their follow-up (difference ≥ 0.05). Three of these four (75%) had reduced FFR values in follow-up (0.91 to 0.8, 0.9 to 0.8, 0.9 to 0.8) and one had a significant increase in follow-up (0.74 to 0.84), respectively. Except for the FFR values, none of the other variables showed a significant difference between those showing a significant change in FFR and those not showing such a difference (Table 2).

**Table 2:** Comparison of characteristics of lesions showing significant change in FFR (>0.05) with those showing no significant change FFR: Fractional flow reserve; AWMI: Anterior wall; IRA: Infarct-related artery; LAD: Left anterior descending coronary artery; RCA: Right coronary artery; LCX: Left circumflex coronary artery; NIRA: Non-infarct related artery; CVA: Cerebrovascular accident; EF: Ejection fraction

Variable	Significant change FFR diff > 0.05 (n = 4)	No significant change (n = 30)	‘p’
Mean Age ± SD	56.75 + 5.38	56.47 + 7.76	0.944
Male	3	21	1.000
Female	1	9	
Smoker	2	12	1.000
Tobacco	2	15	
Hypertension	1	17	>0.05
Diabetes	2	8	
CVA	2	4	
AWMI	2 (50%)	13 (43.3%)	1.000
IRA: LAD	2	13	0.860
RCA	2	15	
LCX	0	2	
Stent Diameter (mm): 2.5	1	5	0.868
2.7	1	11	
3	2	14	
Stent length: <20/21-30/31-40	1	5	0.774
21-30	2	12	
31-40	1	13	
NIRA > 70% stenosis: LAD	1	12	0.783
LCX	2	8	
others	1	10	
Mean lesion length ± SD	21.5 + 6.2	19.7 + 5.42	0.550
Mean lesion diameter ± SD	2.93 + 0.43	2.95 + 0.21	0.856
Mean EF ± SD	46.75 + 7.09	48.73 + 3.27	0.333
Mean FFR ± SD	0.86 + 0.08	0.77 + 0.08	0.042

Out of 34 lesions with >70% narrowing at the first angiogram, 20 lesions had FFR < 0.8. At follow up 23 lesions had FFR < 0.8. There was a change in decision in 5 of 34 lesions (14.7%). Four shifted from medical therapy to invasive strategy and one shifted from invasive to medical therapy.

**Figure 2:**
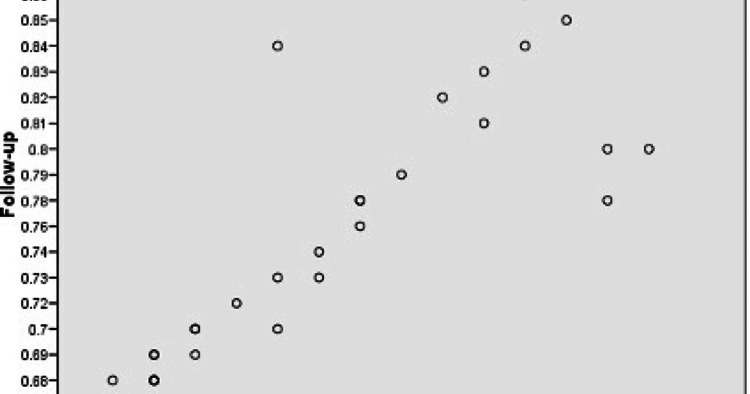
Correlation between baseline and follow-up FFR values Scatter Plot FFR: Fractional flow reserve

A strong positive correlation between baseline and follow-up FFR values was observed (r > 0.7) (Figure 2). The Bland-Altman plot also showed a highly consistent relationship between the mean difference and mean of baseline and follow-up values, thus showing that the change in ejection fraction (EF) and FFR was systematic in nature and could be predicted with high confidence.

## Discussion

The present study indicates that in FFR of NIRA, there is a significant difference at 1-4 weeks after initial FFR done at the time of pharmaco-invasive PCI (mean FFR initial = 0.78 ± 0.08 (0.68-0.93) to mean FFR follow up = 0.77 ± 0.08 (0.67-0.93)) (p = 0.014). We found a major difference in four (11.7%) of our initial values as compared to the follow-up (difference ≥ 0.05). The majority of lesions (three out of four) had significant overestimation of FFR (i.e., underestimation of severity) while one had an underestimation of FFR at the time of initial presentation. Based on the change in FFR values, there was a change in our initial decision in five lesions (14.7%), four of which converted from medical therapy to stenting and one vice-versa. There were no predictors in the demographic profile of patients or lesion characteristics where a significant difference was observed when compared to other lesions, except that mean FFR was higher in patients where major change was observed.

Status and significance of NIRA revascularization

The protocol for the management of multivessel disease amenable to PCI is not defined by any guideline in patients with STEMI undergoing a pharmaco-invasive approach. Significant lesions in non-IRA continue to pose a problem in acute situations. The contemporary practice is to deal with the culprit lesion in IRA at index hospitalization within 24 hours of symptom onset, followed by stage PCI of significant lesion/lesions in other major non-culprit epicardial vessels at a later stage or different setting during the same hospital stay unless the patient has significant stenosis with less than TIMI III flow in NIRA or cardiogenic shock. For the latter, NIRA is also dealt with index procedure [10].

Role of FFR 

Because the angiographic assessment of lesion severity is often misguiding [11,12], the functional assessment of intermediate lesions is traditionally performed by noninvasive testing in the first few days or weeks after pharmaco-invasive PCI or is guided by symptoms of the patients. These tests, however, are expensive and are often difficult to perform or interpret soon after the acute event. The assessment of the functional severity of the non-culprit coronary artery stenosis is clinically important early after pharmaco-invasive PCI to avoid unnecessary delay and complications in the revascularization. For intermediate lesions in stable CAD, the role of FFR has been proven in assessing the physiological significance [13-16] but its role in the setting of ACS has been less well understood. The role of FFR was studied in the Third Danish Study of Optimal Acute Treatment of Patients with ST-Segment Elevation Myocardial Infarction - Primary PCI in Multivessel Disease (DANAMI 3-PRIMULTI) study and it was found that in patients with STEMI and multivessel disease, complete revascularization guided by FFR measurements significantly reduced the risk of future events compared with no further invasive intervention after primary PCI [17]. Similarly, in the recently published COMPARE-ACUTE study by Smits et al., patients with STEMI and multivessel disease who underwent primary PCI of an infarct-related artery, the addition of FFR-guided complete revascularization of non-infarct-related arteries in the acute setting resulted in a risk of a composite cardiovascular outcome that was lower than the risk among those who were treated for the infarct-related artery only. This finding was mainly supported by a reduction in subsequent revascularizations [18].

Controversies regarding FFR use in ACS

To the best of our knowledge, there is no report on a comparison of the initial and follow-up FFR values in NIRA in patients undergoing pharmaco-invasive PCI. The reason for scarce data on FFR in ACS is that FFR values in the culprit vessel have been reported to be higher during acute episodes in comparison to measurements made after the microcirculation has had some time to recover [5,6]. It is postulated that this is due to a reduction in the level of attainable hyperemia in the culprit vessel due to embolization of thrombus and plaque, ischemic microvascular dysfunction, and myocardial stunning [19]. So use of FFR in the setting of ACS remains debatable. The compromised status of vasculature may lead to global changes and alter the FFR findings in distant coronary vessels too.

There is scarce data on the use of FFR in the non-culprit artery for assessing the physiological significance during acute situations [6-7,17-18]. A logical way to validate the FFR values in ACS is to compare serial values of FFR at presentation and a later stage and look for reproducibility or variation. Serial measurement and comparison of FFR in lesions in NIRA in patients of ACS may help in highlighting the effect of hemodynamic and microcirculatory changes on FFR and in establishing the reliability of FFR in this setting. The only study comparing serial FFR values in NIRA has been done in patients with STEMI undergoing primary PCI [20]. Also, the studies evaluating FFR have evaluated its role in intermediate lesions (≈50% stenosis). In patients with ≥70% stenosis, the consensus is to proceed with revascularization. There is a higher probability of FFR to be positive (<0.8) in patients with severe stenosis. There is a need to assess the reliability of FFR values in the population of patients undergoing pharmaco-invasive PCI which is different from primary PCI.

These findings were different from the study by Ntalianis et al. where the population studied was patients undergoing primary PCI. In their study, AWMI was 24% of STEMI while the culprit artery was RCA in 49%. So we had more anterior wall myocardial infarctions with LAD as the culprit artery. In their study, being a primary PCI study, the time from symptom onset to PCI was less than our study (230 ± 201 min). In their study, FFR remained unchanged between acute and follow-up phases in patients with STEMI. Our follow-up catheterization was performed at an earlier stage as compared to their study (mean follow-up duration 35 days) but Ntalianis et al. [20] mentioned in their study that FFR did not change significantly whether the follow-up measurements were performed within seven days or more than seven days after the acute phase. The lesion severity in our patients was greater (mean 78.2 ± 8.69) as compared to their study which had lesser severe lesions (56 ± 14 % DS). They mentioned that in two lesions, the FFR value was higher than 0.8 during the acute phase and lower than 0.75 at follow-up.

So our data indicate that FFR measurements in non-culprit coronary artery stenoses change significantly when measured during the acute phase of an MI and some days or weeks later. These findings suggest that the severity of non-culprit stenoses can not be reliably assessed by FFR during the setting of pharmaco-invasive PCI. In about 15% of lesions, the change in FFR value between the acute phase and the follow-up angiogram led to a change in revascularization strategy. The microvascular dysfunction that had been described in the contralateral territories during the early weeks after an acute MI may have a role in these patients. Also, the patients in our study were followed up earlier, had severe lesions and lower EF when compared to Ntalianis et al., and remained in a longer pose of jeopardy as in the pharmaco-invasive approach. This may have an effect on microvasculature in distant vessels and lead to inaccurate FFR assessment.

However, FFR led to a reduced number of stenting in our patients to approximately 2/3rd of what would have been used if only visual angiography was available.

Study limitations

The patient population was limited and a larger study is required to give robust data for the reliability of FFR. Measurement of FFR prolongs the procedure and increases the contrast use and radiation exposure but the advantage we may achieve is reduced need for noninvasive imaging and repeat catheterization in many patients. The present study was not powered to investigate differences in clinical outcomes.

The results of this study do not suggest that the non-culprit stenoses should not be revascularized during the acute phase of an MI. At present, multivessel revascularization in the acute phase of an MI should be contemplated only in patients with cardiogenic shock and critical non-culprit stenoses.

## Conclusions

The present study indicates that FFR values in NIRA at the time of PCI of culprit lesion in patients undergoing pharmaco-invasive approach are not reliable indicator as compared to follow-up FFR. Though the use of FFR may help in avoiding excess stenting in patients of STEMI with multivessel disease, the value of FFR, however, is not reliable in the acute condition of pharmaco-invasive PCI for assessment of physiological significance of NIRA as the values showed a significant change when repeated within 1-4 weeks. The findings of this study need to be validated in a larger sample size. There were no significant predictors to assess this change in the FFR values barring the initial FFR values.
